# Book Reviews

**Published:** 1910-12

**Authors:** 


### BOOK REVIEWS

THE PRACTICE OF SURGERY. By James G. Mumford, M. D., Instructor in Surgery in the Harvard Medical School. Octavo of 1,015 pages, with 682 illustrations. Philadelphia and London: W. S. Saunders Company, 1910. Cloth, $7.00 net; half morocco $8.50 net.

Mumford has presented a book well adapted to the use of the general surgeon, having avoided the comprehensiveness found in works upon special branches of surgery. He has omitted a special section upon the principles of surgery, but takes up these principles incidentally at appropriate times. Surgical diseases are taken up in the order of interest, importance and frequency, emphasis having been placed upon the subjects which nature herself has accentuated. The student anu practitioner are therefore enabled to see and recognize these affections in their true perspective. With a few exceptions the advice and management of surgical diseases may be highly commended. One of the exceptions is his suggestion as to the value of passage of urethral sounds in the treatment of gonorrhoea. The reviewer is convinced that this pernicious system of "ironing" out the inflamed mucous membrane is the cause of many troublesome and painful complications, such as epididymitis and prostasis and that many otherwise comparatively simple affertions are thus rendered very difficult to cure.

---

AN EPITOME OF HYGEINE AND PUBLIC HEALTH. By George M. Price, M. D., fomerly Inspector New York State Tenement Commission, Medical Sanitary Inspector, New York Department of Health. 12mo. 255 pages. Cloth, $1.00 net. Lea & Febiger, Publishers, Philadelphia and New York, 1910. (Lea's Series of Medical Epitomes. Edited by Victor C. Pedersen, M. D., New York.)

Hygiene may justly be said to be the most important of the medical sciences. The old adage "that an ounce of revention is worth a pound of cure" does not fully state the case, for it remains to be said that preventive medicine deals with the maintenance of the health of thousands, whereas curative medicine renders its benefits to individuals singly. The immensity of the subject and the volumes that have been written on single subdivisions exceed the time and attention which the average reader can bestow, hence the value of an authoritative epitome like the present one. The reader mastering this small volume will be well fitted for examination and for putting his knowledge into practice.

ESSENTIALS OF LABORATORY DIAGNOSIS Designed for students and practitioners. By Frances Ashley Faugh, M. D., Director of the Laboratory of the Department of Clinical Medicine, and Asst. to the Professor of Clinical Medicine, Medico-Chirugical College, Phila. Illustrated. 336 pages. Second Revised Edition. Price $2.00 net. Published by F. A. Davis & Co., Philadelphia.

The second edition of this manual while having been brought up to date, is still of handy size, and renders available in a simplified form the important laboratory tests for the general practitioner. It is by the use of the various tests by the physician himself, who can associate the findings with the clinical symptoms, that the greatest good can come from laboratory methods. Nothing can add so much to accurate diagnoses, satisfactory treatment and increased interest in the practice of medicine as appropriate laboratory tests and there is no reason at all why every physician should not have at least a small laboratory and such a book as the one we have now under review.

---

A TREATISE ON DISEASES OF THE SKIN. For the use of advanced students and practitioners. By Henry W. Stelwagon, M. D., Ph. D., Professor of Dermatology, Jefferson Medical College, Philadelphia. Sixth edition, revised. Handsome octavo of 1195 pages, with 289 text-illustrations, and 34 full-page colored and half-tone plates. Philadelphia and London, W. B. Saunders Company, 1910. Cloth, $6.00, net; half morocco, $7.50, net.

Seven editions of this well known book have been required to supply the demand which in itself is a splendid tribute to its usefulness. The present edition contains much that is new on such subjects as pellagra—the chapter upon this having been wholly re-written—sporotrichosis, grain-mite dematitis, gangosa, tropical ulcers, and ulcerating granuloma. Many improvements in treatment have been recorded.

THE PRACTICE OF MEDICINE. A Guide to the Nature, Discrimination and Management of Disease. By A. O. J. Kelly, A. M., M. D., Asst. Prof. of Medicine in the Univ. of Pennsylvania, etc. Illustrated. 945 paes Lea & Febiger. Philadelphia, Pa.

This book has been prepared by Kelly, who is well known to the medical world through his able and conservative editorship of the best monthly Medical Journal in America, namely, The American Journal of Medical Sciences, to give the essentials of practice of medicine in a clear, concise form for students and practitioners. It appears that his effort not to "violate the physiology of the mind," by crowding into it every possible fact, but to cull them down to useful ones, has enabled him to present to the world a book which should go through many large editions.

Few readers realize how difficult it is for an author to condense medical subjects and yet have the book properly balanced and in good perspective. Kelly has succeeded wonderfully well in this as he has devoted most of the space to the practical phases of medicine. We congratulate the author upon his success and heartily recommend the book.

---

THE ESSENTIALS OF HISTOLOGY, DESCRIPTIVE AND PRACTICAL. For the use of Students. By Edward A. Schafer, F. R. S., Professor of Physiology in the University of Edinburgh. New (8th) edition, thoroughly revised. Octavo, 571 pages, with 645 illustrations. Cloth, $3.50, net. Lea & Febiger, Publishers, Philadelphia and New York, 1910.

Professor Schafer has again thoroughly revised his well-known work. It covers the subject of human histology in a series of fifty short chapters or lessons, systematically arranged and so planned that each suffices for a convenient assignment of classwork. Practical directions are given throughout, so that the work serves alike for didactic and laboratory courses.

